# Hepatitis C Virus-Induced Mitochondrial Dysfunctions

**DOI:** 10.3390/v5030954

**Published:** 2013-03-21

**Authors:** Charlène Brault, Pierre L. Levy, Birke Bartosch

**Affiliations:** CRCL, INSERM U1052, CNRS 5286, Université de Lyon, 151, Cours A Thomas 69424 Lyon Cedex France; E-Mails: charlene.brault@inserm.fr (C.B.) ; pierre-l.levy@inserm.fr (P.L.L.)

**Keywords:** hepatitis C virus, pathology, hepatocarcinogenesis, mitochondria, oxidative stress, metabolism, calcium signaling, apoptosis

## Abstract

Chronic hepatitis C is characterized by metabolic disorders and a microenvironment in the liver dominated by oxidative stress, inflammation and regeneration processes that lead in the long term to hepatocellular carcinoma. Many lines of evidence suggest that mitochondrial dysfunctions, including modification of metabolic fluxes, generation and elimination of oxidative stress, Ca^2+^ signaling and apoptosis, play a central role in these processes. However, how these dysfunctions are induced by the virus and whether they play a role in disease progression and neoplastic transformation remains to be determined. Most *in vitro* studies performed so far have shown that several of the hepatitis C virus (HCV) proteins localize to mitochondria, but the consequences of these interactions on mitochondrial functions remain contradictory, probably due to the use of artificial expression and replication systems. *In vivo* studies are hampered by the fact that innate and adaptive immune responses will overlay mitochondrial dysfunctions induced directly in the hepatocyte by HCV. Thus, the molecular aspects underlying HCV-induced mitochondrial dysfunctions and their roles in viral replication and the associated pathology need yet to be confirmed in the context of productively replicating virus and physiologically relevant *in vitro* and *in vivo* model systems.

## 1. Introduction

Hepatitis C virus (HCV) is a major cause of chronic viral hepatitis with 150–170 million chronic carriers worldwide. Chronic hepatitis C frequently exhibits an insidious course of disease marked by progressive liver injuries that progress, often over several decades, from fibrosis to cirrhosis and, ultimately, hepatocellular carcinoma (HCC) [1]. Spontaneous resolution of infection is rare, and it is estimated that in 80% of cases, HCV establishes a chronic infection that is associated with complex clinical manifestations, including inflammation, insulin resistance (IR) and steatosis. Disease progression and response to therapy are greatly influenced by viral and host genetic factors, including viral genotype and IL28B polymorphisms [2]. Furthermore, risks factors, like alcohol consumption, or metabolic predispositions/diseases, including diabetes, insulin resistance, obesity and steatosis, can aggravate the course and progression of disease [3,4]. The fact that metabolic disturbances and the risk of hepatocarcinogenesis are known to drop back to baseline with successful antiviral response [3] support the notion that metabolic alterations induced by HCV play an important role in viral propagation, as well as the associated pathogenesis. 

HCV is a 9.6 kb-long positive-sense, single-stranded RNA virus of the *Flaviviridae* family. Its genome encodes a polyprotein that is post-translationally cleaved to produce three structural proteins comprising the capsid protein, Core, and the two glycoproteins, E1 and E2, the p7 protein, as well as six non-structural proteins, including the protease, NS2, the helicase and protease, NS3, and its cofactor, NS4A, NS4B, NS5A and RNA-dependent RNA-polymerase, NS5B [5]. Some of the viral proteins have already been demonstrated to interact with and alter cellular signaling pathways [6]. Overall, it has been shown that HCV proteins strongly interact with endoplasmic reticulum (ER)-derived membranes, the site where the viral proteins are synthesized and matured, and induce the formation of “membranous web” structures in which viral replication is thought to occur [7]. HCV proteins have also been localized to the Golgi apparatus and the outer mitochondrial membrane (OMM), and notably, the Core protein, here referred to as Core, is known to be present at the surface of lipid droplets. Particularly, the formation of membranous webs at the ER and the close vicinity of Core to juxtaposed lipid droplets are thought to be important for the HCV assembly process, which is likely to occur at the ER/lipid droplet interface [8] and which remains a much investigated topic.

Mitochondria play a central role in the regulation of metabolic fluxes and the energy status of the cell. They sense and can counteract cellular stress and are the central switch that decides between cell growth, proliferation, differentiation and apoptosis. HCV has been shown not only to associate with, but also to alter, mitochondrial functions and signaling with important consequences for viral replication and the pathogenesis associated with chronic hepatitis C. The interactions between HCV and mitochondria within the hepatocyte and their potential pathological consequences are the topic of this review and are discussed in detail below.

## 2. Mitochondrial Structure and Function

Mitochondria are frequently referred to as "cellular power plants", because they generate most of the cell's supply of adenosine triphosphate (ATP). As sensors of the cells energy status, they decide over cell death, growth, proliferation and differentiation, but are also involved in a range of other processes [9]. Mitochondria have been implicated in a significant number of human diseases, such as cardiovascular disorders, including atherosclerosis, ischemic heart disease, ischemia-reperfusion injury and cardiac failure, as well as neurodegenerative disorders related to mitochondria-derived oxidative stress, such as Huntington’s disease, Parkinson’s disease and Alzheimer’s disease. Finally, mitochondrial functions have been shown to play a role in the aging process and diabetes [10]. 

**Figure 1:**
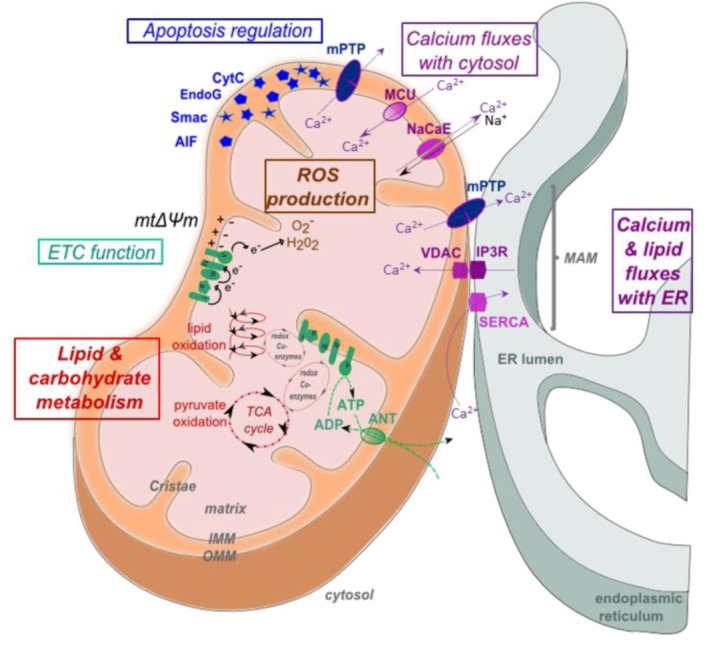
Structure and functions of mitochondria. Mitochondrial membranes and compartments comprise the matrix, inner mitochondrial membrane (IMM), outer mitochondrial membrane (OMM), cristae and mitochondria-associated membrane (MAM). MAMs are points of contact between mitochondria and smooth endoplasmic reticulum (ER), enriched in certain factors, including the inositol triphosphate receptor (IP3R), voltage-dependent anion channel (VDAC) and sarco/endoplasmic reticulum calcium ATPase (SERCA). The main functions of mitochondria are represented in color codes. In red: fatty acids and acetyl coenzyme A (acetyl-CoA) are oxidized by β-oxidation (also called Lynen helix) and the tricarboxylic acid cycle (TCA), respectively, and energy is transferred onto redox coenzymes. In green: the electron transport chain (ETC) is composed of five respiratory complexes (numbered I to V) and uses the electrons of the coenzymes generated by β-oxidation and the TCA cycle as substrates to generate a proton gradient (mtΔΨ) across the inner membrane. The flux of protons from the intermembrane space through complex V (ATP synthase) back into the mitochondrial matrix finally leads to ATP generation. ATP is exported to cytosol via adenine nucleotide translocase (ANT). In brown: electrons that leak from the ETC react with O_2_, forming superoxide anion O_2_^•-^ and hydrogen peroxide H_2_O_2, _also referred to as reactive oxygen species (ROS). In blue: a number of caspase activators are retained in the intermitochondrial membrane space, including, e.g., cytochrome *c*, endonuclease G and apoptosis-inducing factor (AIF). These are released into the cytosol by pro-apoptotic signaling, which leads to opening of the mitochondrial permeability transition pore (mPTP). In violet: mitochondria constantly exchange calcium with the cytosol via the mitochondrial calcium uniporter (MCU) and Na^+^Ca^2+^ exchanger (NaCaE) and with the ER via the mPTP, which comprises, e.g., IP3R and VDAC.

Mitochondria comprise an OMM, which encloses the entire organelle and has a protein-to-phospholipid ratio similar to that of the eukaryotic plasma membrane (Figure 1). The OMM contains porins and translocase complexes that allow diffusion of small proteins or translocation of factors displaying a specific signaling sequence at their N-terminus, respectively. The OMM can associate with the ER membrane via protein tethering complexes, in a structure called mitochondria-associated ER-membrane or MAM [11]. MAMs form discrete junctions where the OMM and inner mitochondrial membrane (IMM) meet, in order to allow an exchange between ER and mitochondria. Indeed, MAMs are responsive to ER stress [12] and vital for transfer of Ca^2+^ and lipid from the ER to mitochondria. MAM fractions are enriched in a number of Ca^2+^ transfer proteins, like sarco/endoplasmic reticulum calcium ATPase (SERCA), which replenishes ER Ca^2+^ stores and inositol triphosphate receptor (IP3R), which together with the voltage-dependent anion channel (VDAC), cyclophilin D and other factors, forms part of the mitochondrial (mt) permeability transition pore (mPTP) and mediates Ca^2+^ transfer between the ER and mitochondria [13]. Furthermore, MAMs appear to be an intermediate destination between the rough ER and the Golgi in the pathway that leads to very-low-density lipoprotein (VLDL) assembly and secretion [14]. The intermembrane space is the space between the outer and the inner membranes. Because the OMM is freely permeable to small molecules, the concentrations of small molecules, such as ions and sugars in the intermembrane space, are the same as in the cytosol. However, because transport of large proteins depends on the activity of translocase complexes in the OMM, the protein composition of this space is different from the protein composition of the cytosol. One protein that is localized to the intermembrane space in this way is, e.g., cytochrome *c*. The IMM is compartmentalized into numerous cristae, which expand the surface area of the IMM and contains proteins that catalyze oxidative phosphorylation, generate ATP in the matrix and regulate metabolite passage and protein trafficking into and out of the matrix, as well as mt fusion and fission. In addition, the IMM is highly impermeable, and almost all ions and molecules require special membrane transporters to enter or exit. The impermeability of the IMM allows the buildup of a membrane potential across the inner membrane (mtΔΨ), which is formed by the action of the enzymes of the electron transport chain (ETC) and which is required for ATP synthesis. The matrix, the space enclosed by the inner membrane, contains a highly-concentrated mixture of hundreds of enzymes, whose major functions include oxidation of fatty acids and the tricarboxylic acid cycle (TCA) cycle.

Mitochondrial energy production is modulated by cell energetic demand, which induces a Ca^2+^ influx into the mt matrix mediated by the Ca^2+^ uniporter (MCU) and Na^+^Ca^2+^ exchanger (NaCaE), which regulate the TCA cycle rate [15]. Indeed, TCA dehydrogenase enzymes and adenosine nucleotide translocase are Ca^2+^ sensitive [16,17,18]. Ca^2+^ fluxes into mitochondria have a major impact not only on metabolism, but also on stress and survival. Indeed, mitochondria can serve as Ca^2+^ buffers, taking up substantial amounts of Ca^2+^ from the cytosol or the ER at the expense of loss of mtΔΨ in order to shape and buffer cellular Ca^2+^ signals. Mitochondrial Ca^2+^ overload, in turn, triggers the opening of the mPTP and induces apoptosis. The fact that mPTP opening is sensitive to antioxidants forms an important link between Ca^2+^ and reactive oxygen species (ROS) [19,20]. 

**Figure 2:**
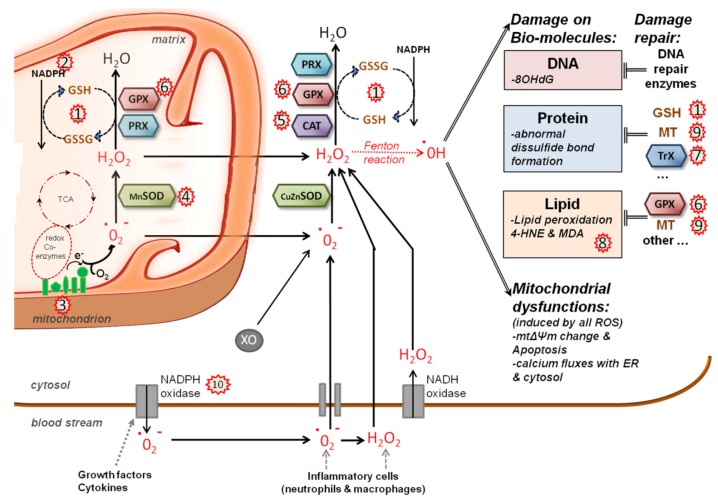
ROS generation and scavenging in HCV infected cells. The main source of mt reactive oxygen species (ROS) is the electron transport chain (ETC); electrons leak from complexes I and IV and produce superoxide anion (O_2_^•-^) and hydrogen peroxide (H_2_O_2_), which is membrane permeable. Non-mt sources of O_2_^•-^ and H_2_O_2_ are cytosolic xanthine oxidase (XO) and plasma membrane NAD(P)H oxidases. The cellular detoxification system scavenges ROS and converts it into H_2_O and O_2_: Mitochondrial (Mn) or cytosolic (Cu/Zn) superoxide dismutases (SOD) degrade O_2_^•-^ into H_2_O_2_, which is then converted into H_2_O and O_2_ by either catalase (CAT), glutathione-peroxidases (GPx) or peroxiredoxin (PRX), which oxidize glutathione (GSH) in the process. Recycling of oxidized glutathione (GSSG) into GSH requires NADPH coenzyme. In addition to GSH, metallothionein (MT) and thioredoxins (Trx) are also used to scavenge ROS peroxidation products. If ROS are considerably augmented, hydroxyl radicals (HO^•^), the most damaging form of ROS, can be produced by the Fenton reaction. ROS producing or scavenging factors whose action is modified by HCV are indicated by red stars and include: 1) oxidation of the glutathione pool [40,60]; 2) decrease in NADPH content [61]; 3) increased ROS production from ETC complex I [40,61]; 4) induction of mitochondrial SOD expression [60]; 5) catalase activation [60]; 6) induction of glutathione peroxidase [49,62]; 7) oxidation of the thioredoxin pool [60]; 8) lipid peroxidation [49,63]; 9) induction of metallothionein [49,64]; and 10) cytoplasmic ROS production by NADPH oxidase activation [65,66].

**Figure 3:**
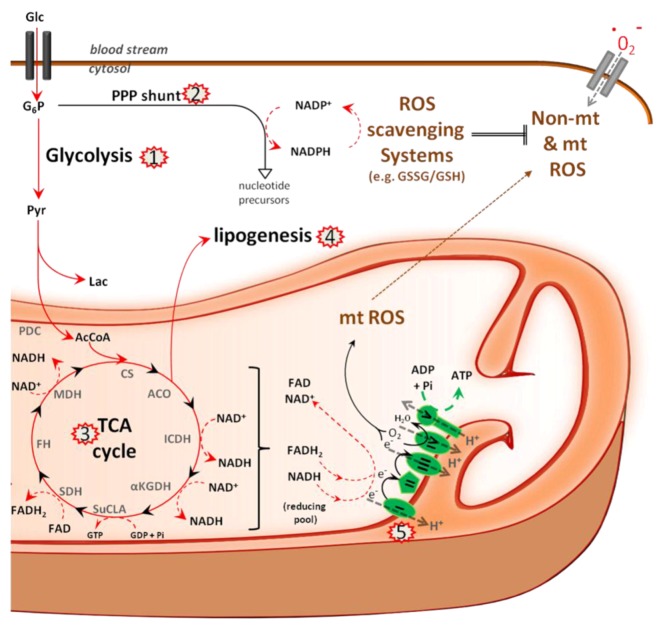
Metabolism in HCV infected cells. The major metabolic pathways that use glucose (Glc) to either drive nucleotide synthesis and NADPH production via the pentose phosphate shunt (PPP) or to drive the TCA cycle and respiration in mitochondria are depicted. TCA reactions catalyze the reduction of NAD^+^ and FAD into NADH and FADH_2_, which in turn feed the electron transport chain (ETC). The ETC maintains the mitochondrial membrane potential, mtΔΨ, required for ATP synthesis. Electrons that leak from complex I and IV of the ETC form reactive oxygen species (ROS). ROS scavenging requires reduced glutathione (GSH), which is oxidized in the process. Recycling of the GSH pool requires NADPH, which is mainly produced by the PP shunt. Glucose-6-phosphate (G6P), pyruvate (Pyr), lactate (Lac), acetyl coenzyme A (AcCoA), pyruvate dehydrogenase complex (PDC), citrate synthase (CS), aconitase (ACO), isocitrate dehydrogenase (ICDH), α-ketoglutarate dehydrogenase (αKGDH), succinyl coenzyme A ligase (SuCLA), succinate dehydrogenase (SDH), fumarate hydratase (FH), malate dehydrogenase (MDH). Factors and events targeted by HCV are indicated by red stars and include: activation of glycolytic enzymes (1) with potential re-routing of the flux into the pentose phosphate shunt, (2) induction of TCA cycle (3) and lipogenic enzymes (4) and complex I of the ETC (5).

The generation of ROS is largely due to the activities of the ETC and mitochondrial dehydrogenases [21] (Figure 2). Under normal conditions, only a small percentage of electrons does not complete the whole ETC and, instead, directly leaks to O_2_, resulting in the formation of free-radical superoxide, O_2_^•-^, for around 0.1% of the O_2_ consumed [22,23]. It has also been shown that mitochondrial Ca^2+^ uptake can lead to radical production, but the underlying mechanism in not understood [24]. Finally, extramitochondrial sources of ROS exist, such as xanthine oxidase or NADPH oxidases [20]. The increase of ROS is interpreted by the cell as an encounter of stress, and ROS are therefore usually immediately eliminated. Indeed, ROS can oxidize proteins, lipids and DNA and, thus, directly contribute to cellular transformation. However, an increase in ROS can also generate numerous physiological responses, spanning from the induction of inflammatory mediators, such as the signal transducer and activator of transcription (STAT) and NFκB pathways, to induced cell death [25]. The detoxification of ROS is an important function of the cellular redox homeostasis system. Cells rapidly convert O_2_^•-^ into H_2_O_2_ with the help of superoxide dismutases (SOD). H_2_O_2_ can then be degraded by a number of enzymes of the cellular redox system, including catalase (CAT), glutathione peroxidases (GPx), thioredoxins (Trx) and peroxiredoxins (PRX). Thus, under normal physiological conditions, ROS are present at low concentrations, which are maintained by a fine balance between Ca^2+^ uptake, mitochondrial energy production, ROS generation, ROS detoxification and redox signaling (Figure 3). 

## 3. Mitochondrial Morphology in Productive HCV Infection

The number, morphology and organization of mitochondria within a cell are correlated with energy needs and health. Indeed, apoptotic cell death is characterized by alterations in mt size, shape and distribution [25]. Several viruses inducing chronic infections have already been shown to alter mt morphology. Clustering of mitochondria is induced by hepatitis B virus (HBV) protein X [26]. In the context of human immunodeficiency virus (HIV), Vpr protein has been shown to condense the mt matrix, shorten mitochondria, induce swollen cristae and disappearance of OMM and degradation of mitochondria-ER contact [27,28]. Human T-cell leukemia virus 1 p13 protein induces mitochondria swelling and fragmentation [29], and expression of the PB1-F2 protein of the influenza virus induces a punctuate pattern of mitochondria organization [30]. Overall, virus-induced changes to the mitochondria network are generally associated with mt dysfunctions [31], but the respective roles of these mt dysfunctions in viral replication and the associated pathologies remain unclear.

HCV infection is known to cause autophagy, ER and oxidative stress and alter Ca^2+^ signaling. It is therefore thought to alter mt structure and functions [32,33,34,35,36,37,38,39,40,41,42,43,44]. Infection of Huh7.5 cells with replicative HCV induces a disruption of the mtΔΨ, followed by mt swelling and cytochrome *c* release [45]. Huh7 cells harboring the full-length genomic, as well as subgenomic replicons, display a decreased number of mitochondria per cell, with a concomitant increase of size [46]. Core or Core-induced ROS were thought, at least in part, to be responsible for these modifications. NS4A protein expression also induces a redistribution of mitochondria in the perinuclear region in Huh7 cells [47]. 

## 4. Physical Interactions between HCV and Mitochondria

Several HCV proteins have been shown to directly associate with mitochondria (Figure 5). Particularly, Core has been shown to associate not only with lipid droplets (LD) [48], but also with mitochondria in Core expressing cells [49,50,51], Core transgenic mice [40], Huh7 cells harboring HCV replicons [47,50] or HCV biopsies [52]. Upon ectopic expression, Core has also been shown to be enriched in MAM fractions on the mitochondrial surface [51]. While initial reports showed that Core associates exclusively to the OMM via a C-terminal motif [40,51], recent electronic microscopy data suggest that Core can also be associated with the IMM [46]. While confocal studies using HCVcc infected Huh7.5 cells did so far not confirm a direct interaction of Core with mitochondria [53,54], biochemical evidence suggests that the interaction does also take place in the context of productively replicating HCVcc [55]. In Huh7 cells expressing p7, NS4A, NS3/4A or harboring a subgenomic RNA replicon, p7 and NS4A were found localized to mitochondria and ER by confocal microscopy, subcellular-fractionation and electron microscopic analysis [47,55,56,57,58,59]. NS5A has been known to be present in particular parts of the ER membrane, so-called “membranous web” structures, where replication is thought to occur [7]. In addition, NS5A is also found on mitochondria and, more precisely, in the matrix and on the IMM [46,47,55]. Similarly, NS5B was recently localized by electron microscopy to the IMM, as well as OMM and the mt matrix [46]. 

## 5. Mitochondrial Functions Altered by HCV Infection

Overall, many lines of evidence suggest that several HCV proteins interact directly with mitochondria in hepatocytes and profoundly alter their functions in metabolism, redox balance, ROS scavenging and apoptosis (Figure 5). However, the molecular mechanisms underlying these direct physical interactions, how these interactions translate into altered mt functions and what roles altered mt functions play in the viral lifecycle and the pathogenesis associated with chronic hepatitis C are issues that are not yet well understood.

### 5.1. Metabolism

Glucose is the major carbon source for the TCA cycle in hepatocytes. It is converted by glycolysis into pyruvate and actively transported across the IMM, converted to acetyl-CoA, which then enters the TCA cycle (Figure 3). The enzymes of the TCA cycle are located in the mt matrix, with the exception of succinate dehydrogenase, which is bound to the IMM as part of electron transfer complex II (see below). The TCA cycle oxidizes the acetyl-CoA to carbon dioxide and, in the process, produces reduced cofactors, NADH and FADH_2_, the substrates for the ETC. The redox energy from NADH and FADH_2_, which is produced not only by the TCA cycle, but also by fatty acid oxidation and amino acid oxidation, is stepwise transferred to oxygen by electron passage through the four complexes of the ETC and used to pump protons into the intermembrane space, thus creating an electrochemical proton gradient across the IMM: the mtΔΨ. Protons then return to the matrix through the ATP synthase complex, and their potential energy is used to synthesize ATP. Generally, only a small percentage of electrons in the ETC form prematurely reduced oxygen, forming reactive oxygen species, such as superoxide. Under physiological conditions, ROS are efficiently scavenged to avoid mitochondrial and cellular oxidative damage. 

In the context of a large scale proteomic study using HCVcc-infected Huh7.5 cells, a number of glycolytic enzymes and enzymes driving the pentose phosphate shunt (PPS) have been shown to be upregulated [33]. However, albeit an increased flux through the glycolytic pathway and a supposed increase in pyruvate production, pyruvate seemed to be used rather for lactate production than for use in the TCA cycle [33]. Furthermore, upregulation of enzymes driving the PPS, such as transketolase and transaldolase, indicates that glycolytic intermediates are channeled into this pathway, which constitutes the major source of NADPH and controls nucleotide synthesis and homeostasis. Indeed, replication in HCV replicon-containing cells is known to depend on sufficient levels of intracellular uridine and cytidine triphosphate [67], and HCVcc-infected cells display lowered ATP levels, while the ATP/ADP and ATP/AMP ratios remained unchanged [68]. However, concomitant with a rechanneling of glycolytic intermediates into the PPS, Diamond *et al.* also observed a coordinated increase in the abundance of TCA cycle enzymes, including citrate synthase, isocitrate dehydrogenase 2 and 3A, fumarate hydratase, malate dehydrogenases 1 and 2 and components of the ETC [33]. These findings suggest that HCVcc-infected cells maintain TCA cycle activity to produce the metabolic precursors for fatty acid synthesis. Indeed, Diamond *et al.* observed the upregulation of lipogenic enzymes, but also an increased abundance of proteins associated with peroxisomal and mitochondrial fatty acid oxidation, suggesting both active lipid synthesis and turnover at the same time. In line with these data, dodecenoyl coenzyme A delta isomerase, a mitochondrial fatty acid oxidation enzyme, has been identified to be required for HCV replication [69]. However, others have reported that HCV decreased expression of the fatty acid-activated transcription factor peroxisome proliferator-activated receptor alpha, which controls β-oxidation and lipoprotein metabolism, as well as its target gene carnitine palmitoyl acyl-CoA transferase 1 gene, which shuttles long-chain fatty acids across the membrane [70,71,72]. Furthermore, addition of a hypolipidemic agent to HCVcc-infected cells that restored β-oxidation and lipogenesis back to normal inhibited replication of the virus, but the contrary has also been reported [69,72]. Thus, it is becoming clear that HCV alters metabolic fluxes through mitochondria; however, the underlying molecular mechanisms remain to be confirmed. Furthermore, the kick-off effects of these changes on the production of oxidative stress and the hepatic redox balance and their roles in viral replication remain unclear. 

### 5.2. Redox System

HCV infection has been associated with oxidative stress and changes in the host’s redox balance, as well as with significant increases in peroxides and oxidative damage *in vivo* (Figure 2). Liver biopsies of HCV patients display elevated levels of 8-oxo-desoxihydroxyguanosine (8-OHdG), a DNA oxidation product, and the lipid peroxides, 4-hydroxy-2-nonenal (4-HNE) and malondialdehyde (MDA) [73,74,75,76,77,78]. Elevated MDA levels have also been detected in sera of chronic hepatitis C patients [79,80,81,82]. Furthermore, HCV-induced HCC is associated with more oxidative stress markers than HBV-induced HCC [75,83]. Generally, oxidative stress markers have been shown to correlate with severity of inflammation, grade of fibrosis and hepatic iron storage markers [75,81], raising the question whether oxidative stress is due to host immunity and iron overload [76,77,84,85,86] or whether direct HCV-host cell interactions also contribute to ROS production. 

Pointing to a direct role for HCV in ROS production, oxidative markers are found in patients with mild, moderate or no liver disease [87]. Furthermore, proteomic profiling has shown an upregulation of antioxidant enzymes at early (F1 to F3), but not late, stages of fibrosis [88]. One of the major sources for intracellular ROS production are mitochondria and particularly leaks in the ETC. Indeed, inhibition of the ETC has been observed to augment ROS generation [21]. In order to prevent oxidative damage and the induction of inflammatory processes, cells rapidly scavenge ROS. Thus, under normal physiological conditions, ROS are present at low concentrations. ROS also regulate the activity of target proteins through the reversible oxidation of critical protein thiols. Drastic increases in ROS can induce mPTP opening, leading to, e.g., cytochrome *c* or Ca^2+ ^release and apoptosis [19,20]. Hepatocarcinogenesis is thought to be driven by permanent elevation of ROS that in turn induces chronic cytokine signaling and lipid, protein and DNA oxidation [6]. 

Several lines of evidence suggest that HCV can directly induce oxidative stress, including various cell lines overexpressing the HCV polyprotein or distinct viral proteins, as well as in cell lines containing replicons or HCVcc-infected Huh7.5 cells [40,49,60,65,89,90,91,92,93,94]. The ETC, in particular, has been identified as an important source of ROS in cells expressing various HCV proteins, as well as Core transgenic mice [32,38,49,61,63,91,95,96,97]. For example, expression of the HCV polyprotein inhibited complex I activity and depolarized the mt membrane, overall leading to an increase in mt ROS production [38]. Core protein expression has been shown to lead to ROS production in Huh7 or CHL cells, as well as in in HeLa and CHO cells using ROS sensitive fluorescent probes, such as DCFDA or DHE [49,55,60,92,94,98]. In addition, oxidative stress markers, like lipid peroxides and 8-OHdG, were increased in cells expressing Core [49,63]. This increase of ROS and peroxides was probably caused by mt dysfunctions for two reasons: it was accompanied by a loss of mtΔΨ and pharmacological inhibition of the ETC, which, however, also affects activity of NADPH oxidases and blocked the effect [49,63]. Mitochondria isolated from mice transgenic for Core, as well as E1 and E2 glycoproteins, also displayed a decrease in NADPH content and increased ROS production from complex I substrates accompanied by reduced activity of ETC complex I [40,61]. Besides production of mt ROS, Core is also involved in ROS production at the plasma membrane by activating NADPH oxidase 4 in human hepatoma cell lines [65,99], and productively replicating HCV has been shown to induce not only NADPH oxidase 4, but also isoform 1 [66]. Concomitantly with the induction of ROS, Core has been shown to alter the mitochondrial redox status. In mitochondria isolated from Core transgenic mice and Core expressing Huh7 cells, oxidation of the glutathione and thioredoxin pools was found to be increased [40,60,91]. Core expression has also been shown to induce the expression of mt, but not cytoplasmic SOD [60], suggesting that HCV generates ROS and, at the same time, strengthens the defense system against oxidative stress. Indeed, Core has been shown to induce metallothioneins and glutathione peroxidases [49,64]. Finally, data on Core-induced modulation of heme oxygenase-1 (HO-1) expression, an enzyme that generates redox active compounds, such as iron, remain contradictory [60,61,98,100,101].

Besides Core, the glycoproteins E1 and E2 are also associated with increased ROS production. E1- and E2-expressing hepatoma cells displayed increased levels of H_2_O_2_, and E1-, but not E2-, expressing cells had increased levels of superoxide anions, as well as lipid peroxides [63,98]. However, at least partially, these effects were mediated through activation of the Nrf2/ARE pathway, which induces the transcriptional activation of a battery of anti-oxidative stress response proteins and enzymes implicated in detoxification and glutathione generation, in ROS-dependent and -independent manners [98]. 

Ectopic expression of the non-structural proteins of HCV is also known to drive ROS production, to lead to the oxidation of thioredoxins and to activate catalase and, to a lesser extent, SOD and HO-1 in Huh7 cells [60]. NS3, for example, has been shown to induce superoxide anion, but not H_2_O_2_ production [63,98,99]. NS3 expression leads furthermore to an accumulation of lipid peroxides in Huh7 cells [63]. NS4B has been implicated in H_2_O_2_ production and activation of the Nrf2/ARE pathway in Huh7 cells, but the underlying mechanisms remain unknown [98]. NS5A expression has been shown to lead to ROS generation in CHL, HepG2 and Huh7 cells [90,96,98,102,103], but levels of lipid peroxides remained normal [63]. NS5A associates with ER membranes and induces Ca^2+^ fluxes from the ER to the mitochondria, which are the likely source of NS5A-induced ROS generation [96,103]. The fact that concomitantly with increased mt Ca^2+^ influx, NS5A decreases ATP generation, implies that infected cells cannot replenish ER Ca^2+^ stores via the SERCA pump [103]. Increased ROS generation and an increase of the total oxidized glutathione levels have been confirmed in the context of HCVcc-infected Huh7.5 cells [93,104]. 

The antioxidants, resveratrol, N-acetyl cysteine (NAC), and vitamins A, C and E were reported to improve replication in the OR6 reporter assay system for genome-length HCV RNA replication in hepatoma-derived HuH-7 cells [105,106,107]. Furthermore, lipid peroxides inhibit HCV replication in cells harboring replicons, and this effect can be reversed by vitamin E treatment [108]. In contrast, alcohol-induced increase of ROS has been reported to boost HCV replication [109,110]. The fact that HCV induces oxidative stress and that *in vitro* replication seems to be sensitive to the effect of antioxidants led to a number of experimental clinical trials, unfortunately, so far, with unclear results. Most clinical trials tested antioxidants in combination with the classical Peg-IFN/ribavirin combination. Frequently, a decrease in oxidative markers and histological inflammation was observed, but rarely a decrease in viral load [111,112,113,114,115]. Overall, the antioxidants seemed to improve liver condition and inflammation and, in some cases, also virological responses. However, it remains unclear whether HCV requires the changes to the cellular redox system for efficient viral replication [116]. 

### 5.3. Calcium Signaling

The close physical association between the ER and mitochondria mediated by MAMs results in Ca^2+^ micro domains at contact points that facilitate efficient Ca^2+^ transmission from the ER to the mitochondria [117]. Ca^2+^ transfer at MAMs requires tight interactions between IP3R, Grp75, VDAC and cyclophilin D, as well as the mtΔΨ-driven MCU at the IMM, which are part of the mPTP complex [15,117]. Regulating ER release of Ca^2+^ at the MAM is critical for cell homeostasis. Sufficient intra-organelle Ca^2+^ concentrations are required to stimulate metabolism by activating pyruvate dehydrogenase, isocitrate dehydrogenase and α-ketoglutarate dehydrogenase, all critical for maintenance of the TCA cycle [16,17]. Prolonged increases of Ca^2+^ can, in turn, interfere with the activity of these enzymes. Because the mtΔΨ depends on the activity of the ECT, which in turn is dependent on TCA cycle activity, interference with any of these processes will induce a substrate imbalance that will lead to the generation of ROS (Figure 3). Also, if Ca^2+^ signaling in the mitochondria passes a certain threshold, the mtΔΨ collapses, and the intrinsic pathway of apoptosis is triggered. The anti-apoptotic factor Bcl-2 has been shown to interact with IP3R to limit Ca^2+^ filling of the ER, leading to reduced efflux at the MAM and preventing collapse of the mtΔΨ post-apoptotic stimuli [118]. 

**Figure 4:**
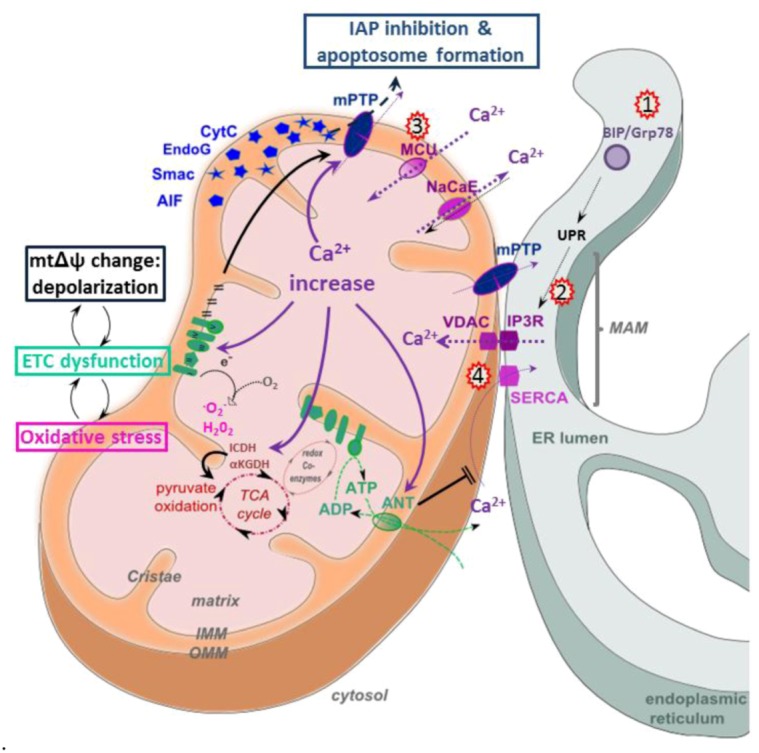
Calcium signaling in HCV infected cells. Mitochondria take up Ca^2+^ via the mtΔΨ-dependent calcium uniporter (MCU), the voltage-dependent anion channel (VDAC), the Na+/Ca^2+^ exchanger (NaCaE) and the mitochondrial permeability transition pore (mPTP). ER Ca^2+ ^uptake is regulated by the sarco/endoplasmic reticulum calcium ATP-ase (SERCA). Ca^2+ ^fluxes are represented by dotted violet arrows. Mt Ca^2+ ^levels control energy metabolism by activating several enzymes of the TCA cycle, such as isocitrate dehydrogenase (ICDH) and α-ketoglutarate dehydrogenase (αKGDH), as well as adenosine nucleotide translocase (ANT), which is a component of the mPTP. Mt Ca^2+^ increase (violet arrows) inhibits ETC function by decreasing mtΔΨ, as well as complex V activity and induces ATP depletion by mPTP opening, leading to the release of pro-apoptotic factors. Kick off effects of Ca^2+ ^increase are marked by black arrows and comprise changes in mtΔΨ, mPTP opening, amplification of ETC inhibition and concomitant ROS generation and ATP depletion. Factors and events targeted by HCV are indicated by red stars and include 1) increase of BIP/Grp78, an endoplasmic reticulum chaperone [35,119], 2) Ca^2+^ transfer from the ER into mitochondria, mediated by NS5A-induced ER stress [96] and by Core expression [55], 3) Core-induced increase of MCU activity [32] and 4) Core-induced SERCA expression [119].

Pharmacological inhibition of ER-mitochondrial Ca^2+^ fluxes in a hepatoma cell line expressing the HCV polyprotein has been shown to normalize all aberrant effects induced by HCV: ETC complex I activity normalized, the loss of mtΔΨ was restored and ROS, as well as intramitochondrial Ca^2+^ concentrations returned to baseline. Time course and titrations of HCV polyprotein expression suggested furthermore that the uptake of Ca^2+^ into mitochondria is the earliest of these above events induced by HCV [38]. Thus, mitochondrial Ca^2+^ uptake may be the initial mt dysfunction induced by HCV and may trigger, in turn, complex I inhibition, loss of mtΔΨ and ROS generation. Another study confirming altered Ca^2+^ fluxes in HCV infection showed that the virus sensitizes Huh7.5 cells to t-butyl hydroperoxide-induced mtΔΨ loss and apoptosis. All these effects could be counteracted by intracellular Ca^2+^ chelation, again suggesting that a dysfunctioning of Ca^2+^ signaling is at their origin [55]. Whether increased mt Ca^2+^ levels in these studies were due to increased mt uptake or release from the ER remained unclear, but another independent study reported Core expressing hepatoma cells to display both increased mt Ca^2+^ entry and transfer of Ca^2+^ from the ER to mitochondria [32]. Finally, NS5A expression has been shown to activate ROS-mediated NFκB and STAT activation, and this process was sensitive to the effects of an MCU inhibitor, suggesting indirectly that alterations of Ca^2+^ fluxes precede ROS production [96,103]. In contrast to the above data, it has also been shown that Core triggers ER stress, including hyperexpression of the stress sensor Grp78/BiP, which in turn leads to mtΔΨ depolarization, cytochrome *c* release and apoptosis. These effects were due to SERCA impairment, followed by ER calcium depletion [119]. Using an ER-targeted aequorin calcium probe, the authors found that ER calcium depletion followed ER stress in Core-expressing cells, suggesting that ER stress was at the origin of these mt dysfunctions [119]. Grp78/BiP overexpression was also confirmed in HCV-infected SCID/Alb-uPA mice [35], but the causal effect on Ca^2+^ signaling was not investigated. Thus, overall, the details and the sequence of the molecular events by which HCV causes cellular stress and alters Ca^2+^ signaling remain unclear. 

### 5.4. Apoptosis

Apoptosis or “programmed cell death” consists of a very well defined series of cellular events that lead to DNA condensation and fragmentation, followed by break down into small apoptotic bodies that are disposed by phagocytes. The intrinsic apoptotic pathway is characterized by mPTP-mediated release of cytochrome *c* or other caspase activating factors, such as Smac/Diablo and HtrA2/Omi from the mt intermembrane space into the cytoplasm. The release of these proteins occurs via the mPTP and is mediated by Bcl-2 family of proteins, whose members display both pro- (Bad, Bax, Bak or Bid) and anti- (Bcl-2, Bcl-xL, Mcl-1) apoptotic properties. Cytochrome *c* then forms a multi-protein complex, known as the ‘apoptosome’, and initiates activation of the caspase cascade through caspase 9. In contrast, the extrinsic apoptotic pathway is activated by ligand binding to death receptors on the plasma membrane, which in turn induces mt cytochrome *c* release or formation of the death-inducing signaling complex (DISC), which activates the caspase cascade through caspase 8 [120]. 

In chronic hepatitis C patients, apoptosis has been suggested to occur in an estimated 0.54% to 20.00% of all hepatocytes based on TUNEL assays and electron microscopy [121,122,123]. However, the strong presence of infiltrating lymphocytes [121,124] and the strong correlation with liver pathology [121,122] and fibrosis grade [125], but not with viral load or genotype, suggest that apoptosis is strongly immune-mediated. Furthermore, with only 10% of hepatocytes estimated to be infected [126,127], it remains unclear whether apoptotic hepatocytes are really HCV infected and whether apoptosis is induced to clear the virus. Thus, so far, the only line of evidence *in vivo* that suggests a direct viral-induced induction of apoptosis comes from SCID mice, in which HCV-infected hepatocytes have been shown to undergo apoptosis [35].

*In vitro*, a direct induction of apoptosis by HCV has been demonstrated in various models and cell lines. Actinomycin D-treated HCV subgenomic replicon harboring Huh7 cells were found to have a decreased Δψm and to undergo higher rates of apoptosis than naive cells. They showed, furthermore, no difference in their sensitivity to TNF-α-mediated apoptosis, suggesting that HCV replication specifically induces mitochondria-mediated cell death [47]. Infection of Huh7.5 cells with the chimeric J6/JFH1 strain led to increased caspase 3 activation and Poly ADP ribose polymerase (PARP) cleavage, accompanied by mitochondrial Bax accumulation, a decreased Δψm and increased cytoplasmic cytochrome *c* [45]. In chimeric SCID/Alb-uPA mice infected with strain H77c, increased rates of apoptosis correlated with increased Bax and decreased Bcl-xL levels [35]. Whether ER stress or the UPR are involved or amplify HCV-induced mitochondria-mediated cell death remains a controversial topic [35,45]. 

Studies focusing on the roles of the individual viral proteins in the induction of apoptosis have particularly identified Core and NS5A as key regulators of ligand-mediated apoptosis, while the role of other viral proteins is still unclear. Core has been demonstrated to possess pro- and anti-apoptotic effects. Core expression induces ligand-independent apoptosis in Jurkat and Huh7 cells by activating caspases 3 [128,129,130], 8 [128] and 9 [129], as well as PARP cleavage [130,131] in SK-HEP-1, 293T or Huh7 cells. In contrast, physiological Core expression in HepG2 cells inhibited caspase 3 activity [132]. Core-induced cytochrome *c* release [129,130] and a loss of Δψm [129] were recorded in 293T and Huh7 cells, strongly suggesting that Core induces apoptosis through the mitochondria-mediated pathway. Moreover, a Bcl-2 homology 3 (BH3) domain has been identified in the Core protein, that may confer pro-apoptotic properties by mediating interaction and inhibition of anti-apoptotic Mcl-1 and cytochrome *c* release into the cytoplasm [130]. Core was also shown to interact with 14-3-3 epsilon protein, leading to Bax release and mitochondrial-mediated apoptosis [129]. In contrast to these data, another study reports that Core only augments TRAIL-mediated apoptosis by enhancing Bid cleavage and activation of the mitochondria apoptosis signaling pathway [133]. Yet, another report shows that Core-induced apoptosis only enhanced death-receptor-mediated apoptosis [134]. Furthermore, the capacity of Core to induce apoptosis seemed to depend on the genotype. Core of genotype 1b was more efficient at inducing apoptosis than Core of genotype 2a, and this was linked to the hydrophobicity of residue 119 [130]. In E1-expressing hepatoma cells, apoptosis depended on the presence of the C-terminal transmembrane domain of E1, presumably altering membrane permeability of E1 [135,136]. E2 has been shown to inhibit death receptor-induced apoptosis in hepatoma cells, presumably through inhibition of mitochondrial cytochrome *c* release [129], and at the same time, it has been shown to induce mitochondria-related and caspase-dependent apoptosis in the same hepatoma cell line [137]. NS2 is known to bind and directly inhibit CIDE-B-induced apoptosis via the mitochondrial pathway [138]. NS3/4A prevents apoptosis by cleaving Cardif, an adaptor protein in the RIG-I antiviral pathway. Upon cleavage, Cardif translocates to the mitochondrial membrane [139], but its precise role in apoptosis induction remains unknown. In contrast, NS3 induces caspase-8 dependent apoptosis in hepatocytes [140] by mechanisms that remain unknown. NS4A expression in Huh7 cells induces cell death, decreases Δψm, induces cytochrome *c* release and caspase 3, but not caspase 8, activation, strongly suggesting that NS4A induces apoptosis through the mitochondria-mediated pathway [47]. NS4B was also found to induce apoptosis via the mitochondrial death pathway in Huh7 cells, where it induced cytochrome *c* release and Δψm decrease, coupled with caspase 3, 7 and 9 activation and PARP cleavage [141]; however, in U-2 OS cells, its action was caspase-independent [134]. NS5A has been shown to inhibit apoptosis by induction of survivin expression, an inhibitor of caspases [142], by blocking cytochrome *c* release [143], by the sequestration of p53 in the cytoplasm to prevent pro-apoptotic gene transcription [144] and by activation of the PKB/Akt and NFkB signaling pathways [96,145]. Bcl-2 homology (BH) domains 1, 2 and 3 were identified in NS5A and the BH2 domain, in particular, has been shown to mediate interaction with Bax, thereby blocking apoptosis [146]. 

**Figure 5:**
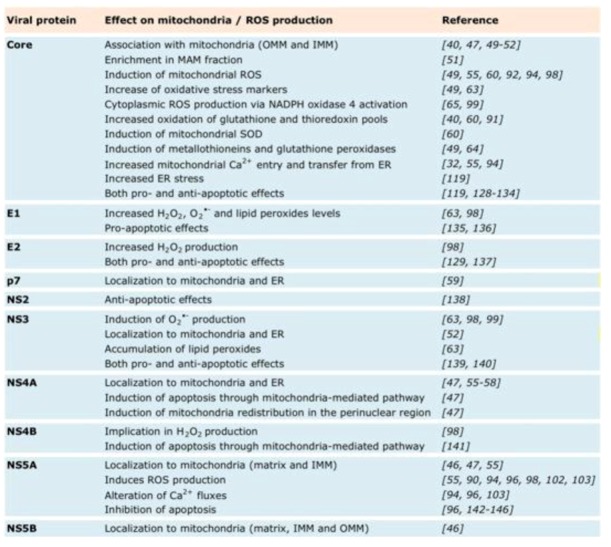
Effect on mitochondria.

## 6. Conclusions

Many viral proteins interact directly with and interfere with signaling pathways that converge in mitochondria. The mitochondrion is the key organelle that determines the cellular response to stress by putting into action stress relief responses, induction of cytokines or apoptosis. Thus, it is not surprising that mitochondrial dysfunctions have been implied in many different forms of diseases. HCV-induced alterations of mitochondrial functions are likely to have a major impact on fibrogenesis and disease progression towards liver cancer by creating a liver microenvironment dominated by oxidative stress and concomitant cytokine signaling and liver regeneration. Restoration of mitochondrial functions is thus an important factor to block and, ultimately, reverse disease progression in chronic hepatitis C, and recent *in vitro* data on the mPTP inhibitor, alisporivir, seem to confirm this notion [147]. However, the molecular details underlying HCV-induced mitochondrial dysfunctions remain confusing and even contradictory at this stage, probably due to the use of very heterologous and artificial *in vitro* expression and replication systems that are based on various different hepatic and non-hepatic cell lines. The lack of an infectious HCV tissue culture system based on physiologically and metabolically relevant cell systems is rendering the investigation of the impact of productively replicating HCV virus on mitochondrial functions *in vitro* very difficult. The possibility to replicate HCV in primary human and murine hepatocytes and the availability of immune-deficient mice with humanized livers will open up new avenues to revisit and validate our existing knowledge and may allow us to use HCV as a tool to better understand the role of mitochondria in other forms of liver disease. 
